# Antibiotic Prophylaxis and Treatment for Cardiac Device Infections

**DOI:** 10.3390/antibiotics13100991

**Published:** 2024-10-18

**Authors:** Claudio Pandozi, Andrea Matteucci, Carlo Pignalberi, Luca Sgarra, Michela Bonanni, Marco Valerio Mariani, Vincenzo Mirco La Fazia, Lorenzo Nesti, Stefania Angela Di Fusco, Federico Nardi, Furio Colivicchi

**Affiliations:** 1 Clinical and Rehabilitation Cardiology Division, San Filippo Neri Hospital, 00135 Rome, Italy; 2 Department of Experimental Medicine, Tor Vergata University, 00133 Rome, Italy; 3 Cardiology Department, Regional General Hospital “F. Miulli”, 70021 Bari, Italy; 4 Fondazione Toscana G. Monasterio, Ospedale del Cuore, 54100 Massa, Italy; 5 Department of Cardiovascular, Respiratory, Nephrological, Aenesthesiological and Geriatric Sciences “Sapienza” University of Rome, 00185 Rome, Italy; 6 Texas Cardiac Arrhythmia Institute, St. David’s Medical Center, Austin, TX 78705, USA; 7 Cardiopulmonary Lab, Department of Clinical and Experimental Medicine, University of Pisa, 56126 Pisa, Italy; 8 Santo Spirito Hospital, 15033 Casale Monferrato, Italy

**Keywords:** cardiovascular implantable electronic devices, CIED infection, antibiotic prophylaxis, antibiotic treatments

## Abstract

Cardiac device infections (CDIs) are a serious complication in patients with implanted devices, resulting in increased morbidity, prolonged hospital stay, and increased healthcare costs. The effective management of these infections involves a combination of appropriate antibiotic therapy and preventive strategies aimed at reducing the risk of infection. The role of antibiotic prophylaxis in infection prevention is crucial, including the emerging use of antibiotic-supported tools and other local antibiotic delivery systems, which may reduce the risk of infection at the device implant site. In this contemporary review, we provide an overview of the prophylactic treatment and different antibiotic regimens for the treatment of CDIs, emphasizing early diagnosis, appropriate choice of antibiotics, and individualized treatment.

## 1. Introduction

Infections associated with cardiovascular implantable electronic devices (CIEDs) are difficult to quantify accurately due to the different patient populations and discrepancies between retrospective and prospective studies with a highly variable incidence of infection [1]. However, two of the most recent prospective studies reported infection rates of 0.6–1.3%, which is substantially lower than previous retrospective studies, suggesting differences in study design and patient selection [2,3]. The clinical spectrum of CIED-related infections is highly variable, ranging from superficial infections at the incision to more severe systemic infections that may involve the pocket or hardware, with or without lead-associated endocarditis. The European Heart Rhythm Association (EHRA) has established criteria to classify these infections, which may manifest as localized infections confined to the surgical site or as systemic forms affecting the heart valves or pacing leads [4]. Systemic infections often lead to more serious complications, especially when vegetations on the tricuspid or pulmonary valve or septic embolism are observed [5]. Given the increasing number and complexity of CIEDs, mainly due to the expansion of indications and the aging population, the severity and incidence of CIED infections are also increasing. Prophylaxis and appropriate antibiotic treatment are critical factors in the management of patient morbidity, mortality, and healthcare costs [6]. Our manuscript aims to analyze the available antibiotic prophylaxis treatment options to prevent CIED infections and, once the diagnosis is established, to determine the appropriate antibiotic treatment according to the specific etiology.

## 2. CIED Infection and Microbiological Investigations

Infections can occur in various forms, which can primarily be categorized as device pocket infections and lead-related infective endocarditis (CIEDs-IE). Although hardware removal remains the definitive treatment for both, distinguishing between the different types of infection and superficial wound inflammation is crucial for clinical management and antibiotic treatment. CIED infections typically present as either localized pocket infections or more severe lead-related endocarditis. Pocket infections are by far the most common and account for approximately two-thirds of all CIED-related infections [7]. They usually occur at the site of the device and are often associated with erythema, warmth, swelling, purulent discharge, and, in severe cases, erosion or exposure of the device. Lead-related endocarditis, although less common, is a more severe condition associated with a higher mortality rate and more complications [5]. It usually involves bacterial colonization of the leads, leading to bloodstream infections and, in some cases, the involvement of the valves. One of the major diagnostic challenges is to differentiate between uncomplicated pocket infections and early postoperative wound inflammation [8]. The latter usually resolves with conservative treatment, whereas the former can progress to a systemic infection if left untreated. Diagnosis is uncertain as both conditions may present with localized erythema and warmth. However, pocket infections tend to manifest beyond the initial wound-healing phase and may be characterized by more prominent signs of infection, such as abscess formation or the development of sinus tracts [9,10]. In contrast, superficial inflammation is usually self-limiting and resolves within the first month after implantation without the need for aggressive intervention [4].

Historically, the modified Duke criteria have been a cornerstone for the diagnosis of infective endocarditis (IE) since their introduction in 2000, as they integrate clinical, microbiological and imaging data. While these criteria have shown an overall sensitivity of approximately 80% for the diagnosis of IE, they have significant limitations, particularly for infections involving implanted devices such as CIEDs, prostheses, or other artificial heart materials. In the context of CIED infections, conventional diagnostic methods, including echocardiography, may not provide clear results. In fact, up to 30% of cases may show normal or equivocal echocardiographic findings even when infection is present [11]. This diagnostic uncertainty is due to several factors, including the poor imaging quality of vegetations on echocardiography due to the presence of metallic or prosthetic materials and the variable clinical presentation of infections associated with these devices. These limitations highlight the need for a more comprehensive diagnostic approach. To address these diagnostic challenges, the European Society of Cardiology (ESC) guidelines in 2015 and, more recently, in 2023 have proposed the inclusion of a multimodality imaging strategy. This improved diagnostic framework integrates echocardiography with advanced imaging techniques such as cardiac and whole-body computed tomography (CT), cerebral magnetic resonance imaging (MRI), 18F-fluorodeoxyglucose positron emission tomography (FDG-PET/CT), and white blood cell single photon emission computed tomography (WBC SPECT/CT) and shows higher diagnostic accuracy, especially in cases where prosthetic material and devices are involved, compared to relying only on the modified Duke criteria [12,13]. FDG-PET/CT has gained considerable attention due to its ability to detect the metabolic activity indicative of infection, particularly in cases of infection involving prosthetic devices where structural abnormalities may not be evident on conventional imaging. The use of FDG-PET/CT has been shown to significantly increase sensitivity in the detection of CIED infections, particularly in cases where conventional echocardiography does not provide clear findings. In addition, WBC-SPECT/CT can provide valuable information by localizing infection sites based on the distribution of labeled white blood cells, further contributing to the diagnostic process in complex cases [14,15]. The recent update to the ESC guideline for the management of endocarditis incorporates the current modified diagnostic criteria of infective endocarditis [5,16] and ranks the likelihood of infection based on these criteria at admission and during follow-up (Table 1 and Table 2).

Microbial identification enables the choice of an appropriate antibiotic therapy. The most common pathogens responsible for CIED infections are coagulase-negative staphylococci (42–77%) and *Staphylococcus aureus* (10–30%). Methicillin-resistant staphylococci, which include both coagulase-negative staphylococci and *Staphylococcus aureus*, account for almost a third of all cases and pose a major challenge for treatment due to their resistance to standard antibiotic therapies [4,17]. *Staphylococcus aureus* infections are of particular concern, especially when they occur early after device implantation, as they are associated with a more aggressive course and an increased likelihood of lead-related endocarditis. These infections often result from contamination during the implantation procedure and can lead to severe systemic infections if not recognized and treated immediately.

Blood cultures are essential for the diagnosis of CIED-related bloodstream infection, especially in cases where patients present with systemic symptoms such as fever, chills, or malaise. Recurrent or persistent bacteremia, particularly in patients with an implantable cardioverter defibrillator (ICD) or a history of prosthetic heart valves, is highly suggestive of CIEDs IE [5].

The infection onset timing of infection provides important clues regarding its origin. Infections that occur within the first year of device implantation are often associated with contamination from the procedure and usually involve the pocket and, in some cases, the leads. In contrast, infections that manifest later are more likely to be due to hematogenous spread from other sources, such as the skin, oral cavity, respiratory tract, or gastrointestinal tract. In some cases, the infection may spread retrogradely from the bloodstream into the pocket, further complicating the diagnostic process [8]. Understanding the routes of infection is critical to developing an appropriate treatment strategy, as the origin of the infection may determine the need for hardware removal versus prolonged antibiotic therapy alone. Blood cultures are essential for the diagnosis of lead-related endocarditis and should be obtained prior to initiating antibiotic therapy.

## 3. Antibiotic Prophylaxis

Prophylactic administration of antibiotics is a proven strategy to reduce the risk of infection associated with CIEDs and is considered standard clinical practice [4]. Numerous studies have shown that the use of prophylactic antibiotics can significantly reduce the incidence of device-related infections compared to no antibiotic treatment, with relative risk reductions ranging from 40% to 95% [18,19]. To achieve optimal tissue concentrations at the surgical site, antibiotic treatment should be started within one hour before the skin incision. The most frequently identified pathogen is *Staphylococcus aureus* with a high prevalence of methicillin resistance [17]. In view of this prevalence, prophylactic antibiotics must offer protection against *Staphylococcus aureus*. However, current evidence does not recommend routine prophylaxis against methicillin-resistant staphylococci. Its use depends primarily on the local prevalence rates of methicillin-resistant staphylococci and the patient’s individual risk factors. Intravenous flucloxacillin (1–2 g) and first-generation cephalosporins, such as cefazolin (1–2 g), provide effective protection against methicillin-susceptible *Staphylococcus aureus*. In cases where the patient has a documented allergy to beta-lactam antibiotics, vancomycin (15 mg/kg) is a suitable alternative. Since vancomycin must be administered over a prolonged period of time (usually an infusion over approximately 60 min is required), it should be administered 90 to 120 min prior to the surgical incision to ensure adequate tissue penetration at the time of the procedure. In addition to the recommended prophylactic administration of antibiotics, good hygiene practices must be applied to prevent surgical infections.

Based on current evidence, the intraoperative irrigation of the device pocket with antibiotics is generally not recommended, nor is the routine postoperative administration of antibiotics. This approach is supported by the results of the PADIT study, which showed that additional perioperative antibiotic therapy did not significantly reduce infection rates in CIED procedures [2]. In patients at an increased risk of infection, the use of advanced prevention strategies, such as antibiotic-releasing envelopes, may provide additional protection. These envelopes, which consist of an antibiotic mesh, release minocycline and rifampin locally for at least seven days, with complete absorption occurring over approximately nine weeks. This tool has been shown to be effective in reducing infection rates in certain patient groups. The WRAP-IT trial, a large-scale randomized study, confirmed the efficacy of these antibiotic envelops and showed a significant decrease in CIED infection rates in patients undergoing high-risk procedures such as pocket revisions, generator replacements, system upgrades, and CRT defibrillator implantations [3,20]. Gentamicin-impregnated collagen sponges are similar products that can also be used for the prophylaxis of CIED infections [21]. Preclinical studies on the local administration of gentamicin have shown its concentration-dependent efficacy on gram-negative bacteria and partially the same effects for gram-positive bacteria, such as staphylococci. This means that higher concentrations of gentamicin lead to faster elimination of bacteria, even in strains with low susceptibility or resistance to the antibiotic. The gentamicin-impregnated collagen implant was also effective in the presence of antibiotic-resistant *Staphylococcus epidermidis* [22]. This efficacy is probably due to the high local concentrations of gentamicin. Local gentamicin delivery has also been proven to be effective in preventing surgical site infection in various types of surgery (e.g., cardiac, orthopedic, and gastrointestinal surgery) without systemic exposure to the drug. The high concentrations achieved locally indicate a rapid eradication of the bacteria in the most important species. While maintaining high gentamicin serum levels may promote the development of resistance, achieving high peak concentrations at the surgical site with minimal systemic exposure may help prevent the emergence of resistant bacteria [23]. Delivering gentamicin directly to the site of infection may help to minimize the associated risks. In contrast, the systemic administration of gentamicin poses notable dangers, including the potential for kidney toxicity and hearing damage.

## 4. Antibiotic Treatment of CIED Infection

The primary approach for treating CIED infection involves early and complete removal of the entire device system, with antibiotics serving to eliminate residual infections in the native tissues [24]. Currently, there are no randomized controlled trials that specifically guide the selection of antibiotics for CIED infection [25]. Table 3 summarizes the antibiotic recommendations for the primary types of CIED infection: superficial incisional infection, isolated device pocket infection, and systemic infections. Obtaining a wound culture before initiating antibiotic therapy is recommended for superficial incisional infections.

The empirical antibiotic regimen should cover *Staphylococcus aureus*, with adjustments made based on the culture results. In the case of isolated pocket infections, intravenous antibiotic therapy should begin after blood cultures have been obtained. Treatment should then be refined based on the culture results, ideally using a narrow-spectrum antibiotic, such as a beta-lactam. Combination antibiotic therapy is not required in these cases. Switching to oral antibiotics is reasonable following device removal since the remaining infection typically involves only the skin and soft tissues. When isolated pocket infections are accompanied by positive blood cultures but without vegetations on the leads or valves, the treatment recommendations align with those for isolated pocket infections, but without switching to an oral antibiotic regimen. For patients with positive blood cultures showing vegetation on the device leads or heart valves, treatment should follow established guidelines for infective endocarditis after pathogen identification [4,5].

Initial broad-spectrum therapy typically includes a combination of ampicillin, cloxacillin, ceftriaxone, or vancomycin with gentamicin. This regimen is maintained until the pathogen identification is confirmed to optimize treatment efficacy. When streptococci are isolated, the recommended treatment for native valve endocarditis (NVE) usually consists of ceftriaxone combined with gentamicin for four weeks, while in prosthetic valve endocarditis (PVE), the treatment duration is extended to six weeks because of the increased risk and complexity associated with prosthetic materials. In staphylococcal NVE infections, the options include flucloxacillin, cefazolin, or vancomycin, with a treatment duration of four to six weeks. In cases of PVE, a combination of flucloxacillin or vancomycin with gentamicin and rifampin for six weeks is recommended to address the biofilmogenic nature of staphylococci on prosthetic valves. The enterococcal species, on the other hand, require regimens for both NVE and PVE that include ampicillin with gentamicin, vancomycin with gentamicin, or ampicillin with ceftriaxone, with a treatment duration of six weeks. Daptomycin has demonstrated comparable efficacy to vancomycin. However, it should be administered at elevated doses (10 mg/kg) and in conjunction with other antibiotics, such as beta-lactams or fosfomycin, to mitigate the risk of developing resistance. This reflects the organism’s inherent resistance patterns and the need for high-level bactericidal activity. In cases where cultures are negative, the treatment approach is determined by a multidisciplinary team, reflecting the complexity and variability in such cases [5,26].

Despite the absence of direct device involvement, device removal may be reasonable in cases where specific pathogens are detected, or if bacteremia recurs without an identifiable source of infection. This occurs in particular for *Staphylococcus aureus*, coagulase-negative staphylococci, cutibacterium species, candida species, beta-hemolytic streptococci, or enterococcus species. In contrast, infections caused by many gram-negative bacteria, such as non-pseudomonas or serratia species and pneumococcal infections, are less likely to involve the device, which can be saved. For patients where complete device removal is not possible, salvage therapy involving long-term suppressive antibiotic treatment is necessary, including intravenous antibiotics for 4 to 6 weeks. The duration of suppressive therapy must be tailored to the individual patient’s clinical needs [27]. For oral streptococci, *Streptococcus gallolyticus* (*S. bovis*) and pyogenic groups sensitive to standard penicillin dosing, a short course of antibiotics lasting just two weeks can be effective. This typically involves a combination of penicillin or ceftriaxone with an aminoglycoside, such as gentamicin administered as a once-daily dose in the case of normal renal function. If an outpatient antibiotic therapy is chosen as feasible, a regimen of ceftriaxone alone or in combination with gentamicin administered once daily is considered especially practical. Patients with a penicillin allergy require desensitization to penicillin/cephalosporins or vancomycin use. It is important to note that beta-lactams are generally considered more effective than glycopeptides. An alternative that has been suggested in some cases is teicoplanin, which should be initiated with a loading dose of 6 mg/kg every 12 h for three days, which is necessary to bind extensively to serum proteins and penetrate vegetation, followed by a maintenance dose of 6–10 mg/kg once daily [5]. For cases where penicillin resistance is confirmed, it is crucial to administer aminoglycosides for a minimum duration of two weeks. Short-term treatment regimens are generally avoided due to the resistance profiles. After two weeks of therapy, clinically stable patients should be evaluated to determine if outpatient parenteral antibiotic therapy or outpatient oral antibiotic therapy is a viable option. The infections caused by *Enterococcus faecalis* that demonstrate resistance to both penicillin and gentamicin pose significant challenges due to the high levels of resistance to aminoglycosides. The recommended regimen is a combination of high doses of ampicillin and ceftriaxone, which enhances the activity of ampicillin by binding to penicillin-binding proteins. The duration of therapy in these cases tends to be longer, typically ranging from 6 to 8 weeks, in comparison to the shorter regimens used for infections caused by penicillin-susceptible organisms. The regimens for *Enterococcus faecium* include vancomycin or teicoplanin. Furthermore, daptomycin, despite its potency, requires high doses (often 10–12 mg/kg/day) to achieve bactericidal activity against these resistant strains, raising concerns about potential side effects, including myopathy. Linezolid, an oxazolidinone-class antibiotic, could be another option; however, it is primarily bacteriostatic, not bactericidal, meaning it inhibits the growth of bacteria rather than killing them outright, which is suboptimal for treating CIED infection. Other experimental treatments include quinupristin–dalfopristin and tigecycline, though their efficacy is limited, and they are generally considered second-line or salvage therapies [28].

## 5. Discussion

The incidence of CIED infection remains a significant clinical challenge despite advances in preventive strategies. A critical issue in CIED infection is the increasing prevalence of methicillin-resistant staphylococci, including both coagulase-negative staphylococci and *Staphylococcus aureus*. These organisms account for up to one-third of CIED infection, making their management challenging due to resistance to commonly used antibiotics [29]. Among the antibiotics used in such contexts, daptomycin, a cyclic lipopeptide, has used for its bactericidal activity targeting multidrug-resistant gram-positive bacteria, such as methicillin-resistant *Staphylococcus aureus* and vancomycin-resistant *Enterococcus faecalis*, both of which are increasingly encountered in hospital settings [30]. Daptomycin has also been used in combination with ceftriaxone for the empirical treatment of CIED infection in patients who underwent device removal, showing a good safety and effectiveness profile in follow-ups [31]. The diagnosis of systemic CIED-associated infection, particularly when local signs of inflammation are absent, is often difficult and can result in significant delays in initiating the correct treatment, sometimes extending for weeks or months. Coagulase-negative staphylococci, such as *Staphylococcus epidermidis*, are common in this setting. As biofilm-forming organisms that are part of the skin’s natural flora, they are particularly prone to causing foreign body infections, similar to *Staphylococcus aureus*, but tend to lead to more subacute or chronic infection due to their lower virulence [26].

The current guidelines advocate for the complete removal of devices and leads, not only for systemic infections but also for localized pocket infections. However, lead extraction can be associated with serious complications, especially in high-risk patients. A recent study on continuous, in situ targeted, ultrahigh concentration of antibiotics offers a potential alternative to device extraction in managing CIED infection. With this new regimen, pocket infections were treated in 85% of patients, suggesting that targeted antibiotic delivery directly into the infected site can be particularly effective for patients where extraction poses prohibitive risks due to the comorbidity of high frailty. Moreover, while extraction may be more effective in preventing infection recurrence, the in situ-targeted, ultrahigh concentration of antibiotics does not negatively impact patient survival [32]. Interestingly, a recent survey conducted among European cardiologists from 18 countries highlights a critical gap in the consistent application of risk assessment tools for CIED infection. While the majority of physicians adopt prophylactic measures, such as intravenous antibiotics and minimizing the number of leads, the use of additional strategies, like antibacterial envelopes, is less uniform [33]. Evidence suggests that improving CIED infection prevention requires not only broader adherence to prophylactic guidelines but also the development of more refined and sensitive risk stratification tools. The fact that a significant portion of respondents expressed the need for clearer guidelines and enhanced scoring systems underscores the limitations of current strategies in addressing infection risks comprehensively.

Several studies have explored the role of prophylactic measures beyond systemic antibiotics [34,35,36]. The WRAP-IT trial [3], a large-scale randomized study, demonstrated the efficacy of antibiotic-releasing envelopes in reducing infection rates in high-risk CIED patients, such as those undergoing generator replacements or system upgrades, releasing minocycline and rifampin locally over a sustained period. Options for antibiotic envelopes include the previously described non-biologic envelope impregnated with rifampin and minocycline, biologic matrix support impregnated with gentamicin, or hydration of the biologic envelope in saline containing one or more antibiotics. These envelopes can be biologic (made from decellularized extracellular matrix) or non-biologic. A recent study assessed the real-world use of biologic antibacterial envelopes during CIED implantation, focusing on how physicians hydrated these envelopes (with or without antibiotics) and how this impacted infection outcomes [37]. A significant reduction in infection rates was observed when biological envelopes were hydrated in antibiotic solutions, particularly those containing gentamicin. This evidence highlights the importance of multi-faceted infection prevention strategies in CIED procedures. A major limitation to the wide use of envelopes is their high cost, potentially confining their use to selected cases with high-risk patients.

Numerous studies address the financial impact of CIED infection on healthcare costs. The reported average costs range from EUR 11 thousand in Poland [38] to EUR 22 thousand in France [6] and up to EUR 35 thousand in the United Kingdom [39]. In the United States, the costs are even more marked, exceeding twice those of the European countries [40]. It is important to note that many of these findings are derived from national healthcare databases, which often provide only aggregate costs without distinguishing between local versus systemic infections or accounting for varying initial treatment strategies. Defining high-risk patients could better address the use of antibiotic tools available to prevent infection which can be carried out using scores [2,41,42]. However, there is currently no shared consensus for their routine use. Local antibiotic delivery systems, such as gentamicin-impregnated collagen sponges, have shown potential in reducing the incidence of surgical site infections in various types of surgeries, including those involving CIEDs [21,43]. Studies have demonstrated that high local concentrations of gentamicin can effectively eradicate both gram-positive and gram-negative bacteria, including antibiotic-resistant strains of *Staphylococcus epidermidis* [44]. This is particularly relevant in cases where systemic administration of gentamicin may be contraindicated due to its nephrotoxic and ototoxic effects. The localized delivery of gentamicin directly to the infection site minimizes systemic exposure, thereby reducing the risk of developing antibiotic resistance while maintaining efficacy against key pathogens. Due to their lower cost than other systems, they could be a useful alternative to more expensive tools, especially in high-risk patients. The analysis of the largest court available to date, involving approximately three thousand patients matched with and without the use of gentamicin-impregnated sponges, estimates that the number needed to treat (NNT) is 105 patients to prevent a single CIED infection [21]. Since patients are not a selected high-risk population, it is conceivable that these numbers would be significantly lower when used in patients with the appropriate criteria already having been discussed. If properly employed through suitable patient selection and the informed use of these tools, the outcomes could improve further. Future studies need to explore, with randomized and long-term follow-up, the outcomes of using local antibiotic delivery systems in CIED patients, particularly in terms of reducing infection-related morbidity and mortality. In addition, while antibiotic prophylaxis remains the cornerstone of infection prevention in CIED procedures, other preventive measures need further study before they can be recommended, such as the effectiveness of postoperative antibiotic therapy following CIED procedures. The current evidence suggests that continuing antibiotic prophylaxis beyond 24 h does not significantly reduce infection rates or improve other outcomes, such as mortality or pocket hematoma rates [45]. Additionally, the practice of pocket irrigation during CIED implantation using antibiotic solutions also calls for more targeted studies. Despite common use, recent data indicate no significant differences in infection rates between saline, bacitracin, or vancomycin irrigation solutions [46]. This lack of clear benefit from antibiotic irrigation underscores the need for rigorous trials to establish best practices. Finally, the development of new antibiotics or alternative antimicrobial agents that can overcome the challenges posed by multidrug-resistant organisms is critical. The growing threat of antimicrobial resistance in CIED infection, particularly with the widespread use of prophylactic antibiotics, necessitates continued research into novel therapeutic options. Recently, new opportunities have emerged for the use of various nanomaterials used for targeted drug delivery to the sites of infection, avoiding systemic effects. Among them, nanoparticles made of silver, copper, and other metals can inhibit biofilm formation through damaging the bacterial cell membrane by increasing its permeability [47]. Another mechanism they exploit involves the generation of reactive oxygen species, which inhibits protein expression and affects replication activities. Nanocarriers that are used as drug delivery systems are characterized by a hollow structure that can deliver drugs by prolonging their half-life, reducing the frequency of administration and adverse effects. Studies on the combinations with amoxicillin and copper nanoparticles against *Proteus mirabilis* or polymyxin B with silver particle nanocarriers against *Pseudomonas aeruginosa* have shown good safety and efficacy profiles [48]. Against the *Staphylococcus aureus* biofilm, there is evidence of enhanced antimicrobial activity by combining antibiotic therapy with nitric oxide-releasing nanoparticles, suggesting that these nanoparticles may have applications in the treatment of infections of the native valve, prosthetic valves, and pacing leads [49]. The integration of antimicrobial stewardship programs within healthcare settings may help mitigate the overuse of antibiotics and slow the emergence of resistant pathogens.

## 6. Conclusions

The prevention and management of CIED infection continue to be a complex and evolving field. While the current guidelines provide a framework for prophylactic antibiotic use, the growing prevalence of antibiotic-resistant organisms presents challenges that require new solutions. The adoption of advanced imaging techniques, local antibiotic delivery systems, and novel preventive devices such as antibiotic-releasing envelopes represents a promising approach to reducing infection rates. However, ongoing research is needed to refine these strategies and address the limitations of current diagnostic and therapeutic approaches. A comprehensive, patient-centered approach that integrates preventive strategies, prompt diagnosis, and targeted therapy is essential in reducing the burden of CIED infection.

## Figures and Tables

**Table 1:** Criteria of infective endocarditis according to 2023 ESC guidelines [5].

Major Criteria	Definition
Blood cultures positive for IE	(a) Typical microorganisms consistent with IE from two separate blood cultures: Oral streptococci, *Streptococcus gallolyticus*, HACEK group, *S. aureus*, *E. faecalis*. (b) Microorganisms consistent with IE from two separate positive blood cultures: ≥2 positive blood cultures of blood samples drawn ≥12 h apart. All 3 or a majority of ≥4 separate cultures (first and last samples drawn ≥1 h apart). (c) Single positive blood culture for *Coxiella burnetii* or phase I IgG antibody titre >1:800.
Imaging positive for IE	Valvular, perivalvular, periprosthetic, and foreign material lesions characteristic of IE, detected by: Echocardiography (TTE and TOE). Cardiac CT. [18F]-FDG-PET/CT(A). WBC SPECT/CT.
Minor Criteria	
Predisposing conditions	Predisposing heart conditions at high/intermediate risk of IE or people who inject drugs (PWIDs).
Fever	Temperature > 38 °C.
Embolic vascular dissemination	Major systemic and pulmonary emboli/infarcts, abscesses, hematogenous osteoarticular septic complications (spondylodiscitis), mycotic aneurysms, intracranial ischaemic/hemorrhagic lesions, conjunctival hemorrhages, Janeway’s lesions.
Immunological phenomena	Glomerulonephritis, Osler nodes, Roth spots, rheumatoid factor.
Microbiological evidence	Positive blood culture but not meeting a major criterion.

**Table 2:** Likelihood of infection based on modified criteria [4].

Definition	Criteria
Definite CIEDs Pocket or Generator Infection	Signs include swelling, redness, warmth, pain, purulent discharge, or visible device erosion.
Definite CIEDs-IE	Requires two major criteria (e.g., positive blood cultures for typical microorganisms or echocardiographic evidence of vegetations) or one major criterion plus three minor criteria (such as fever or vascular phenomena).
Possible CIEDs-IE	One major criterion and one minor, or three minor criteria suggest a potential infection.
Rejected CIEDs-IE	Absence of the aforementioned diagnostic criteria rules out infective endocarditis.

**Table 3:** Antibiotic treatment according to primary types of CIED infection.

Type of Infection	Empirical Treatment	Adjustments	Duration
Superficial incisional infection	Oral antibiotic treatment covering *S. aureus* Flucloxacillin oral or amoxicillin–clavulanate If high MRSA prevalence: trimethoprim–sulfamethoxazole, clindamycin, doxycyclin, linezolid	To be adjusted after culture result	7–10 days
Isolated pocket infection (negative blood cultures)	Methicillin-resistant coagulase-negative staphylococci and *S. aureus*: vancomycin or daptomycin If systemic symptoms: for additional gram-negative coverage, combine with 3rd-gen cephalosporin or gentamicin	To be adjusted after culture result	10–14 days post-extraction
Systemic infections (without vegetation on leads or valves ± pocket infection)	Vancomycin or daptomycin + 3rd-gen cephalosporin or gentamicin If sensitive staphylococci: flucloxacillin or 1st-gen cephalosporin	To be adjusted after culture result	4 weeks (2 if negative blood culture)
CIEDs endocarditis with vegetation on leads and/or valves ± embolism	Vancomycin or daptomycin + 3rd-gen cephalosporin or gentamicin If prosthetic valve: add rifampicin after 5–7 days	To be adjusted after culture result	6 weeks or longer
